# Oncotically Driven Control over Glycocalyx Dimension for Cell Surface Engineering and Protein Binding in the Longitudinal Direction

**DOI:** 10.1038/s41598-018-25870-2

**Published:** 2018-05-15

**Authors:** Erika M. J. Siren, Rafi Chapanian, Iren Constantinescu, Donald E. Brooks, Jayachandran N. Kizhakkedathu

**Affiliations:** 1 Centre for Blood Research, Life Sciences Centre, University of British Columbia, Vancouver, BC V6T 1Z3 Canada; 2 Department of Chemistry, Life Sciences Centre, University of British Columbia, Vancouver, BC V6T 1Z3 Canada; 3 Department of Pathology and Laboratory Medicine, Life Sciences Centre, University of British Columbia, Vancouver, BC V6T 1Z3 Canada

## Abstract

Here we present a simple technique for re-directing reactions on the cell surface to the outermost region of the glycocalyx. Macromolecular crowding with inert polymers was utilized to reversibly alter the accessibility of glycocalyx proteoglycans toward cell-surface reactive probes allowing for reactivity control in the longitudinal direction (‘z’-direction) on the glycocalyx. Studies in HUVECs demonstrated an oncotically driven collapse of the glycocalyx brush structure in the presence of crowders as the mechanism responsible for re-directing reactivity. This phenomenon is consistent across a variety of macromolecular agents including polymers, protein markers and antibodies which all displayed enhanced binding to the outermost surface of multiple cell types. We then demonstrated the biological significance of the technique by increasing the camouflage of red blood cell surface antigens via a crowding-enhanced attachment of voluminous polymers to the exterior of the glycocalyx. The accessibility to Rhesus D (R_h_D) and CD47 proteins on the cell surface was significantly decreased in crowding-assisted polymer grafting in comparison to non-crowded conditions. This strategy is expected to generate new tools for controlled glycocalyx engineering, probing the glycocalyx structure and function, and improving the development of cell based therapies.

## Introduction

The glycocalyx is a highly complex, glycoprotein-rich region of the extracellular matrix on the cell surface. Directly attached to the cell membrane, the glycocalyx has a myriad of reported regulatory functions^1^. As such, it is a useful target for controlling cellular behavior and techniques which engineer the glycocalyx and other aspects of the cell surface have emerged in response. Using macromolecules such as polymers, enzymes, antibodies and proteins, this technique has had great success in modulating cellular response and function^2–5^. For instance, various forms of cell-surface engineering have been carried out to control stem cell differentiation^6^, in targeted drug delivery using T and B cells to treat tumors^4^, glycoengineering of tumors for targeted drug delivery^7^ and tPA-carrying red blood cells (RBCs) for selective dissolution of nascent blood clots^8^.

In developing cell surface engineering methodologies, the goals are divergent depending on the desired outcome. Fundamental investigations of the cell surface often require site specificity, and subsequent surface-targeted genetic and metabolic engineering techniques have been developed to employ substrates with site-specific biorthogonal tags^9–11^. Alternatively, in the pursuit of redirecting of cellular behavior or delivering therapeutics it is desirable to maximize the amount of agent delivered to the cell surface^12–15^. In these circumstances, simple chemistry is often used to modify the often abundant thiol or lysine moieties of the existing glycocalyx structure^4,16^.

When attaching macromolecules to the cell surface, both applications require a large stoichiometric excess of functionalized substrate to impart a cellular response^17,18^. The use of excess cell interactive macromolecules is not only costly, but can also cause unwanted side effects and toxicity^19–21^. As the glycocalyx is the first contact point for effectors in solution, we hypothesize that directing modification to the outermost region could enhance the effect of the attached substrates – be it immunoevading polymers^22–24^, scaffolds for tissue engineering^25–27^, or cell homing substrates^28–30^. Thus far, tools which enable controlled modification in the *longitudinal* (‘z’) direction of the glycocalyx are largely undeveloped and selectivity along this axis remains indiscriminate even in site-specific approaches.

Herein, we present a technique to reversibly alter the accessibility of glycocalyx proteoglycans toward cell-surface reactive probes in solution. We show that the use of inert macromolecular crowders in cell media can reversibly collapse the glycocalyx and enhance the binding of polymers, protein markers and antibodies to the structures’ outermost surface of multiple cell types. This methodology can be used to discriminately probe glycocalyx function in the ‘*z*’ direction or amplify the biological response of surface engineered cells, as we demonstrated in the improved immunocamouflage of engineered red blood cells.

## Results and Discussion

### Probing crowded-assisted redistribution of polymer grafts on the cell surface

**Figure 1:**
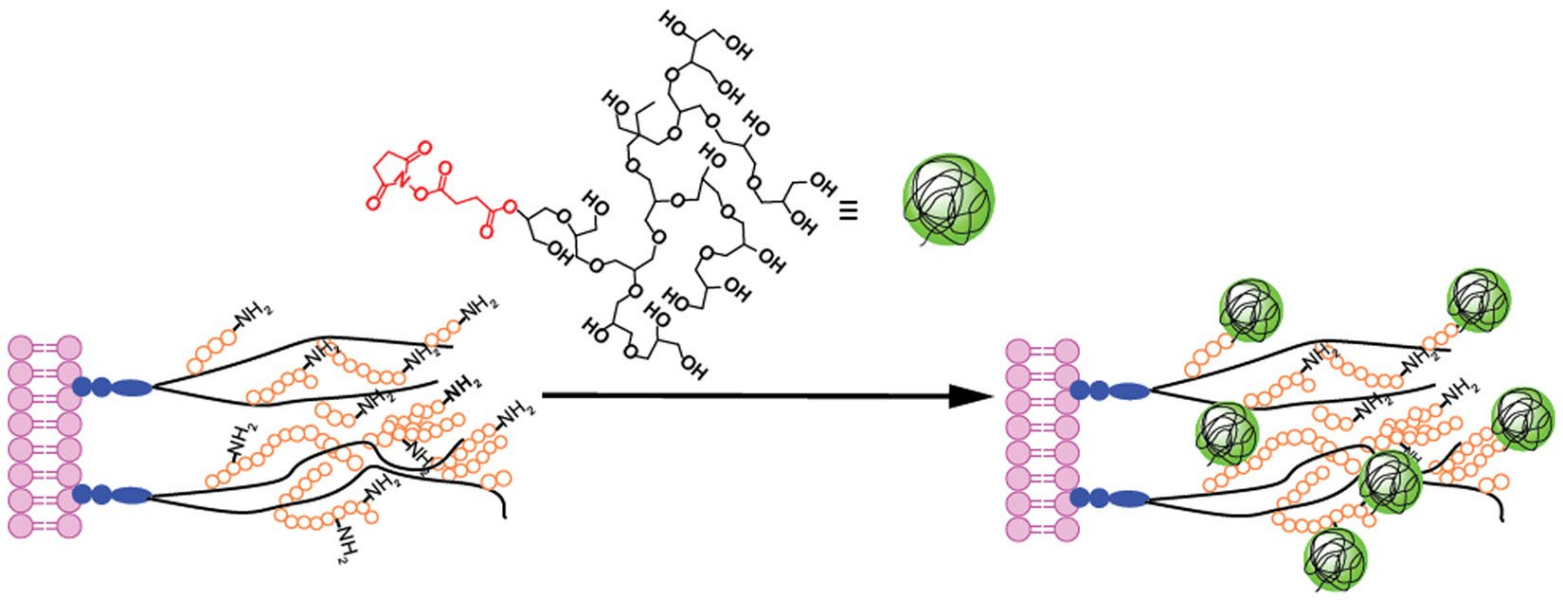
Strategy for ligation of polymers to primary amines on the glycocalyx using succinimidyl esters (red).

For cell surface modification studies, we employed amine-reactive polymer molecules which target lysine residues on the cell surface glycocalyx (Fig. 1)^22,23^. Succinimidyl succinate (SS) modified hyperbranched polyglycerol (HPG-SS, 20 kDa) was used to covalently couple hyperbranched polyglycerol (HPG) to the lysine residues on cell surface proteoglycans. It should be noted that SS modified substrates are not specific for the glycocalyx residues. When these substrates are used for cell surface engineering applications however, the components of the extracellular matrix (including the glycocalyx) are often preferentially modified as they are the first point of contact for protein-reactive substrates in the bulk solution. This was verified by measuring the decrease in polymer present on the cell surface when the glycocalyx was specifically removed enzymatically (Fig. 1S, Table 1S). A corresponding decrease in the intensity of glycocalyx labelling (Alexa-633 conjugated wheat germ agglutinin (WGA)) was observed suggesting the preferential grafting of polymer on glycocalyx. For generating temporary crowded conditions, unreactive HPG (30 kDa) was used as a macromolecular crowder at a 230 mg/mL dissolved in PBS solution. While a series of macromolecular agents may be used to impart crowding conditions (i.e. PEG, ficoll, dextran), the superior cell compatibility of HPG elicited by the polymer’s compact nature was the reasoning for its selection in the presented study^31^.

**Figure 2:**
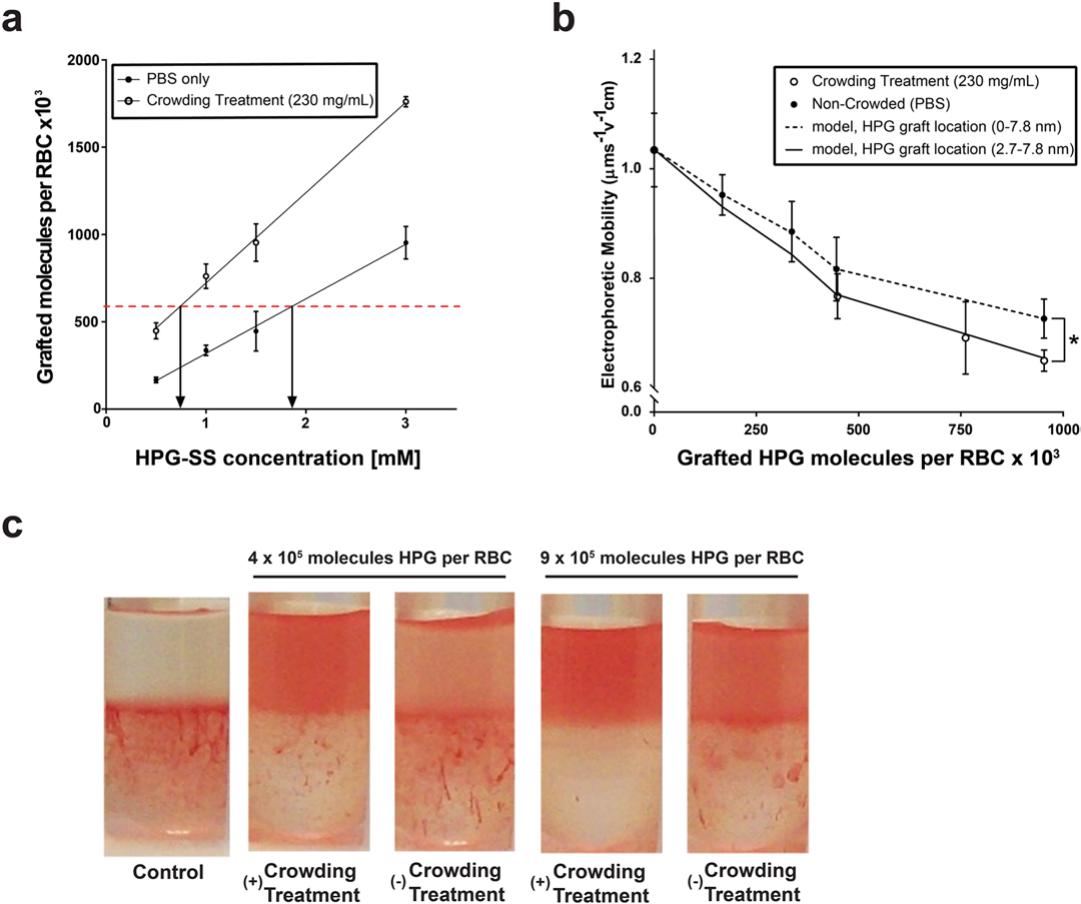
Changes in the physical parameters of RBC’s following polymer grafting under crowded and non-crowded conditions. (**a)** Impact of macromolecular grafting method (non-crowded vs crowding treament) on the number of HPG molecules grafted per RBC, quantified using the tritium (^3^H) radiolabeled HPG. (**b)** Electrophoretic drag consequential to increased polymer grafting on cell surface. Circles represent experimental data of RBC movement in an electric field. Lines represent mathematical simulation to predict electrophoretic mobility of RBCs when polymer molecules differ in their distribution along the glycocalyx. (**c)** Partitioning of HPG-grafted RBCs in PEG-dextran aqueous two-phase partitioning system. Control RBCs are in the lower dextran phase, and those grafted with HPGs move towards the upper PEG phase. Unpaired comparisons using a non-parametric t-test are significant at *p* < 0.05 (*).

Previous work from our group has demonstrated that macromolecular crowding enhances the extent of polymer mediated attachment to the cell surface^12^. To exclusively study the distribution of HPG-SS within the cell surface glycocalyx, it is essential to identify grafting concentrations which produce a similar number of polymer grafts on the cell surface in both conditions (crowding treatment vs. non-crowded). Tritium labelled SS-HPGs (^3^H-SS-HPG) were prepared and used for determining the number of polymer grafts with high accuracy (Fig. 2a). The dotted red line in Fig. 2a represents HPG-SS concentrations which yielded a similar number of HPG molecules on the cell surface under both crowded and non-crowded grafting conditions. These adjusted concentrations were repeated in all future experiments with HPG-SS.

We next investigated the physical characteristics of HPG-modified RBC suspensions which may be influenced by differences in polymer distribution on the glycocalyx. These experiments were performed on washed RBCs to remove the polymer crowder from the solution following polymer attached in crowded and non-crowded conditions. Brooks *et al*. have previously demonstrated that the movement of RBCs in an applied electric field (electrophoretic mobility) is dependent on surface charge density (σ), the thickness of glycocalyx (β), the Stokes effective mean radius of a glycocalyx segment (a), and total mass on the cell surface^32,33^. Their work further illustrated that the attachment of voluminous HPG polymers to the RBC surface should increase the hydrodynamic drag on electroosmotic flow by changing the glycocalyx parameters. Through covalent attachment to the glycocalyx, the polymers extend the Stokes effective mean radius of the glycocalyx elements surrounding the cell and reduce the electrophoretic mobility. Upon measuring the electrophoretic mobility of RBCs grafted with a similar number of HPG molecules, it was demonstrated that cells modified in crowded conditions have a lower mobility compared to those grafted in PBS (Fig. 2b). This result indicates that the grafted polymers impact the glycocalyx parameters in a unique manner when grafted in crowded conditions. With all other controlling parameters for electrophoretic mobility remaining constant, the diminished mobility for RBCs prepared in crowded conditions are consistent an increased Stokes effective mean radius on the glycocalyx which elicits a larger hydrodynamic drag on the modified cells. As both RBC surfaces possess a similar number of HPG molecules, polymers grafted under crowded conditions must be preferentially placed on the exterior of the glycocalyx where the impact on extending the mean radius of the glycocalyx is more pronounced.

We further fitted the experimental data using a mathematical model (solid lines shown in Fig. 2b) developed to predict the electrophoretic mobility of RBCs bearing adsorbed or covalently bound polymers by Brooks and co-workers (details of the calculations are given in supporting information)^32,33^. Using a hypothetical glycocalyx thickness of 7.8 nm, we modeled glycocalyx modification of elements located both throughout the glycocalyx (0.0–7.8 nm) and directed to the outer surface (2.7–7.8 nm). Our analysis indicated that more hydrodynamic shielding is exerted by the HPG grafts when modification is weighted towards the exterior of the glycocalyx. As seen in Fig. 2b, the modelling matches well with the trends observed in measured electrophoretic mobilities.

The properties of grafted RBCs were further investigated using cell partitioning in aqueous two-phase PEG/dextran system; an assay sensitive to changes in the surface properties of RBCs^34,35^. We have shown previously that HPG grafted RBCs partition more towards the PEG phase in a manner that is dose-dependent with increasing polymer concentration^12^. As the cell surface becomes saturated with grafted polymers, the solution phase-polymer interaction begins to dictate partitioning instead of the native RBC surface alone. The greater partitioning of modified RBCs to the PEG phase is due to more energetically favorable associations between the grafted polymer and the PEG-rich phase compared to the dextran-rich phase. As shown in Fig. 2c, RBCs grafted with HPG in crowded conditions partitioned more to the upper PEG phase compared to those prepared in non-crowded conditions. As the number of molecules grafted per cell is similar in both conditions, it can be inferred that HPG grafted in crowded conditions is occurring preferentially on the more solution-phase accessible outer surface of glycocalyx.

### Location of grafted polymer on the glycocalyx

**Figure 3:**
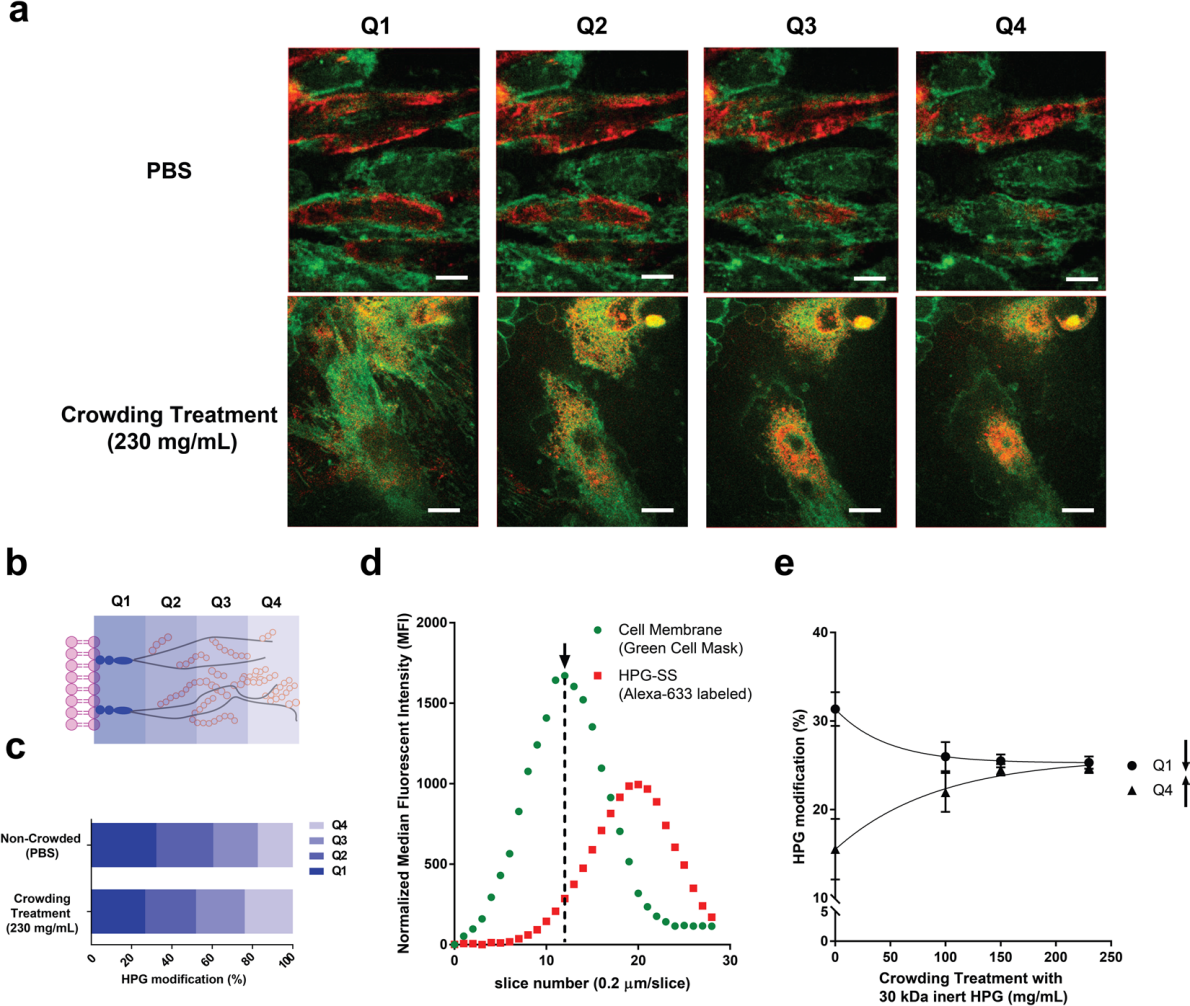
Distribution of fluorescently labeled HPG molecules grafted on the endothelial surface glycocalyx in non-crowded and crowded conditions. (**a)** Representative live cell confocal microscopy z-slices of Alexa Flour-63 labeled HPG (red) modified HUVEC surfaces. The plasma membrane is represented in green. Scale bar represents 10 µm. (**b)** Illustration of the segmentation borders used to study glycocalyx modification in a location specific manner. (**c)** The distribution of Alexa Fluor-633 labeled HPG-SS on HUVEC surfaces prepared under crowded and non-crowded conditions determined from median fluorescent intensities. Percent HPG modification refers to the fraction of HPG found in each quadrant compared to the total amount of HPG grafted along the entirety of the glycocalyx segment. (**d**) Intensity profiles of signals corresponding to the cell membrane (green) and the polymer labelled glycocalyx along the z-axis. The arrow denotes maximal intensity of the cell membrane stain. Intensity measurements in the red channel in slices after this point were compiled and separated into quadrants. (**e)** Influence of crowder concentration (30 kDa inert HPG) on the redistribution of grafted HPG from Q1 to Q4.

The data presented in the Fig. 2 demonstrate that polymer grafting in either PBS or crowded conditions provides cell populations which vary in their surface characteristics. While these effects support changes in grafting distribution, direct visualization is difficult within the small 7–8 nm thick RBC glycocalyx^32,36^. To visualize any changes in graft location on the glycocalyx, we repeated crowding-assisted HPG-SS attachment on human umbilical vein endothelial cells (HUVECs) monolayers. *In vitro*, the endothelial glycocalyx reaches heights of 2–4 microns in the *z*-dimension, almost 500-fold larger than the structures found on the RBC cell surface^37,38^. With a larger glycocalyx structure, high resolution confocal microscopy may be used to directly measure the distribution of HPG along the entirety of the glycocalyx^39,40^. Using Alexa Fluor-633 fluorescently labelled HPG-SS, we modified HUVEC surfaces under varying concentrations of inert crowder. Images were then collected along the *z*-axis (0.2 µm slices) with a high-resolution spinning disk confocal microscope (Fig. 3a). HUVECs were incubated with Alexa Fluor 633 labelled HPG for 1 hour at 4 °C followed by staining of the cell membrane with CellMask^TM^ green plasma membrane stain.

The thickness of the endothelial glycocalyx is heterogeneous not only between cells but around the surface of a single cell^37,41,42^. With this, absolute *z*-values could not be used for comparing the location of HPG along the glycocalyx structure. To more effectively compare cell populations, the total thickness of the glycocalyx beginning from the cell membrane was divided into four quadrants with quadrant one (Q1) being closest to the cell membrane and quadrant four (Q4) being the most apical (Fig. 3b). To account for heterogeneity in total cell height and volume, intensity measurements for Alexa-633 labeled HPG were only taken above the cell membrane. The starting point for all measurements were slices immediately following the slice where cell membrane is in focus and at maximal intensity (Fig. 3d, denoted by an arrow). Using this segmentation pattern, it was demonstrated that the grafting of fluorescently labelled HPG-SS on HUVECs was weighted toward the exterior of the glycocalyx under crowded conditions. (Fig. 3c). With increasing crowder concentration, we observed a dose dependent shift in HPG occupancy (based on median fluorescent intensity) from Q1 to Q4 (Fig. 3e). In a similar fashion to RBC modification, the endothelial cell model demonstrated that crowding-assisted polymer grafting shifts HPG modification from the base of the glycocalyx to the outer surface of the brush structure, exhibiting controlled localization in the *z*-direction.

### Mechanism for redirecting cell surface engineering

We next investigated the biophysical mechanism responsible for enhancing modification to the outermost glycocalyx surface. Under physiological conditions, the highly hydrated proteoglycan brush structures are believed to exist in an extended conformation which supports increased lateral movement and flexibility relative to their anchor points on the cell membrane^43^. The flexible nature of the hydrated brush structure allows for a majority of the reactive residues on the glycocalyx to be exposed to the solution, enabling uniform accessibility to surface reactive agents upon incubation. The hydration of the proteoglycan structures is also a significant contributor to perceived glycocalyx thickness. We postulated that the presence of high concentrations of hydrophilic crowder (230 mg/mL) may promote dehydration-mediated glycocalyx collapse. Earlier work on macromolecular crowding has demonstrated that proteins become compressed in crowded conditions due to the existence of an osmotic pressure gradient in the microenvironment around proteins^44,45^. Macromolecules like albumin and HPG behave like colloids in solution and contribute to the colloid osmotic, or oncotic, pressure of the system when present in sufficient concentrations^46,47^. Previous studies have measured albumin concentrations within the endothelial glycocalyx as 25–50 mg/ml *in vitro*^48^. The introduction of a comparatively high concentration of colloid into the system has the potential to disrupt oncotic homeostasis and establish an oncotic gradient between the glycocalyx and the surrounding crowder solution. As the intricate mesh-work of the glycocalyx behaves like a molecular sieve, the expulsion of water molecules is favored over the diffusion of colloids into the glycocalyx^43^. To re-establish an oncotic equilibrium, water molecules are transferred from the hydrated glycocalyx to the crowding solution – effectively dehydrating and partially collapsing the brush structure. With the innermost region of the glycocalyx being inaccessible to large molecules, it was hypothesized that this collapse may drive preferential modification on the outer surface.

**Figure 4:**
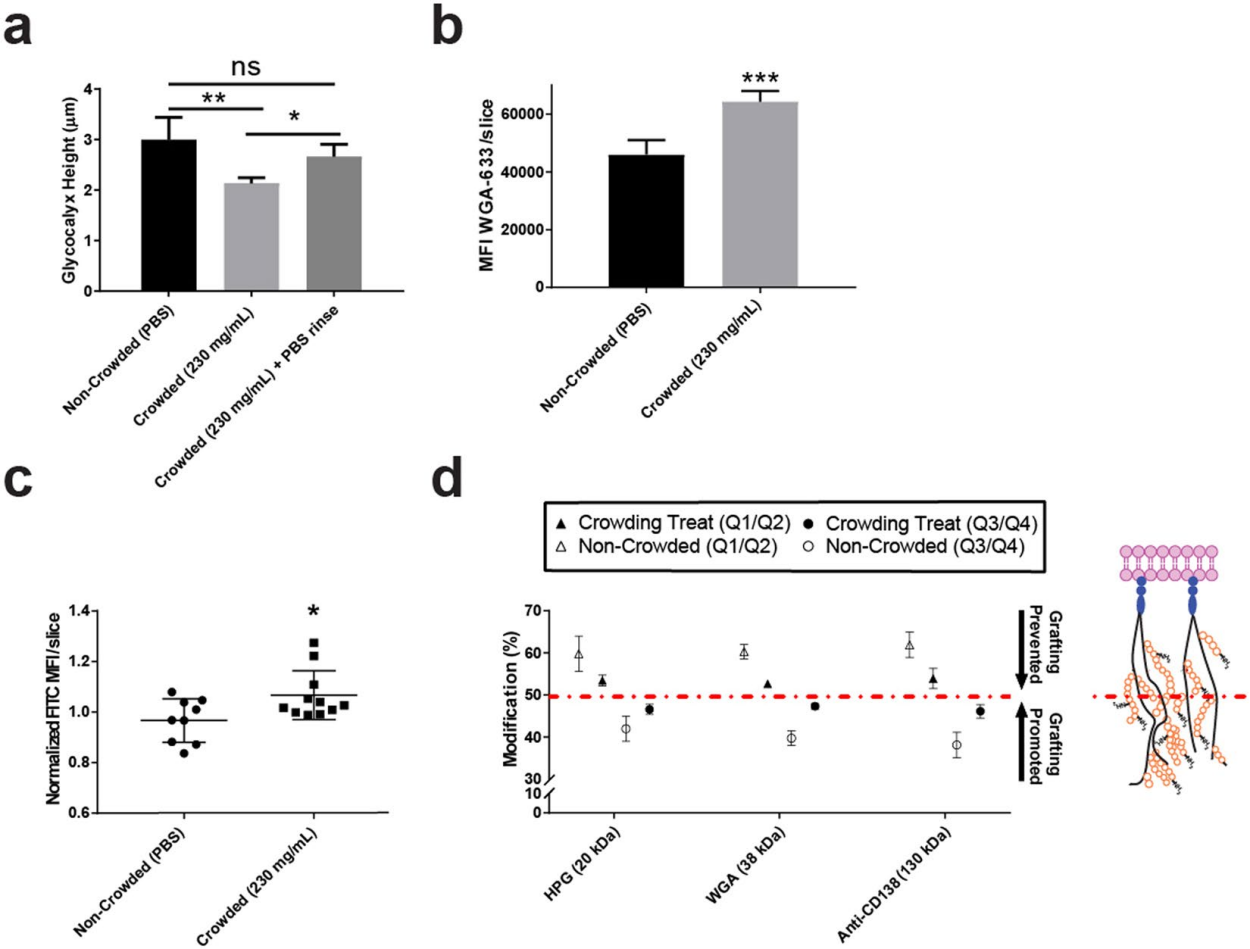
Investigation of the collapse of the endothelial (HUVEC) glycocalyx under crowded conditions. (**a)** Observed changes in height of glycocalyx before, during and after the addition of macromolecular crowder to HUVEC monolayers. (**b)** Density of the glycocalyx in presence and absence of macromolecular crowder. WGA intensity values were normalized to cell number and are presented as a ratio against Hoescht nuclear stain (**c)** Dehydration of glycocalyx under crowded conditions measured using FITC labeled dextran (40 kDa). Values are listed as median fluorescent intensity (MFI) of FITC per z-slice captured using confocal imaging. (**d)** Evidence of the redistributive effect of various cell surface binding macromolecular agents (fluorescent tag labelled polymer, lectin and antibody) from the inner (Q1/Q2) to outer (Q3/Q4) region of the glycocalyx in crowded conditions demonstrating generality of the phenomenon. The dashed red line is used to separate data from the modification of the inner (above) and outer (below) regions of the glycocalyx. Representation of Q1 to Q4 of glycocalyx is given in Fig. 3b. Paired comparisons using a non-parametric t-test are significant with *p* < 0.05 (*) *p* < 0.01 (**) and *p* < 0.001 (***).

**Figure 5:**
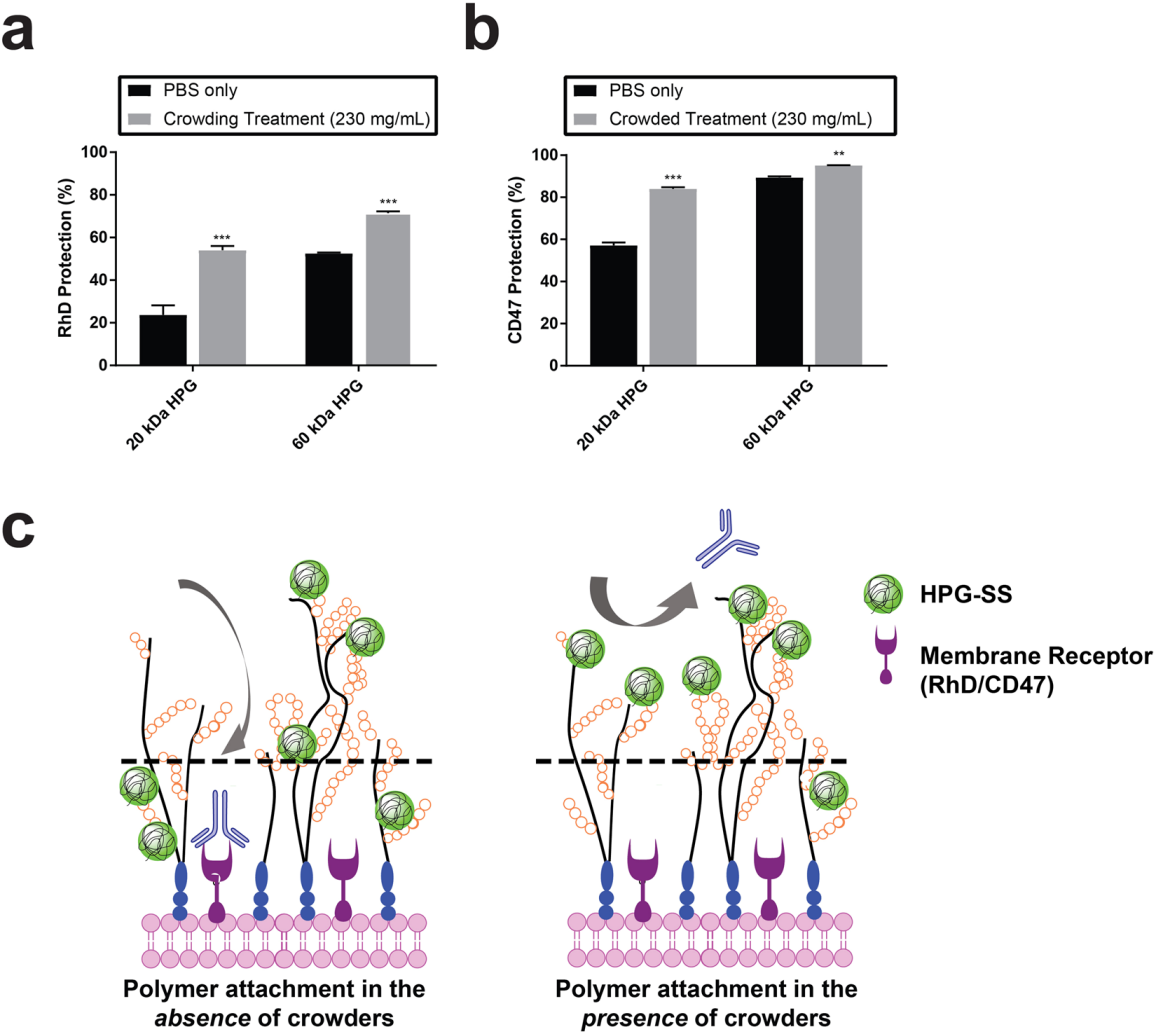
Biological significance of cell surface engineering directed to the outermost surface of the glycocalyx. The impact of the redistribution was demonstrated by measuring the extent of immunocamouflage of cell surface antigens on HPG grafted red blood cells in crowded and non-crowded conditions. The number of grafted HPG molecules on the RBCs are 9.5 × 10^5^ and 9.7 × 10^5^ per cell for 20 kDa and 60 kDa HPG-SS respectively under both crowded and non-crowded conditions. (**a)** Rhesus D (R_h_D) and (**b)** CD47 antigens on the surface of RBCs modified with sterically shielding HPGs. Relative protection compared to unmodified RBC’s was evaluated using flow cytometry analysis and FITC labeled antibodies to R_h_D and CD47. The grafting of polymer molecules preferentially on the outer surface of the glycocalyx aided by the crowded conditions generated a significant increase in the shielding effect of grafted polymer molecules. Unpaired comparisons using a non-parametric t-test are significant, *p* < 0.01 (**) and *p* < 0.001 (***). (**c)** Polymer grafting under crowded conditions forms a polymer shield on the cell surface that is better at camouflaging underlying RBC membrane receptors R_h_D (**a**) and CD47 (**b**). It is proposed that this enhanced effect is on account of polymer grafting concentrated to the apical region of the RBC glycocalyx where grafted polymers are more adept at preventing the access of Ab in solution to the membrane bound receptors. Dashed lines in figure separate the upper and lower regions of the glycocalyx.

**Figure 6:**
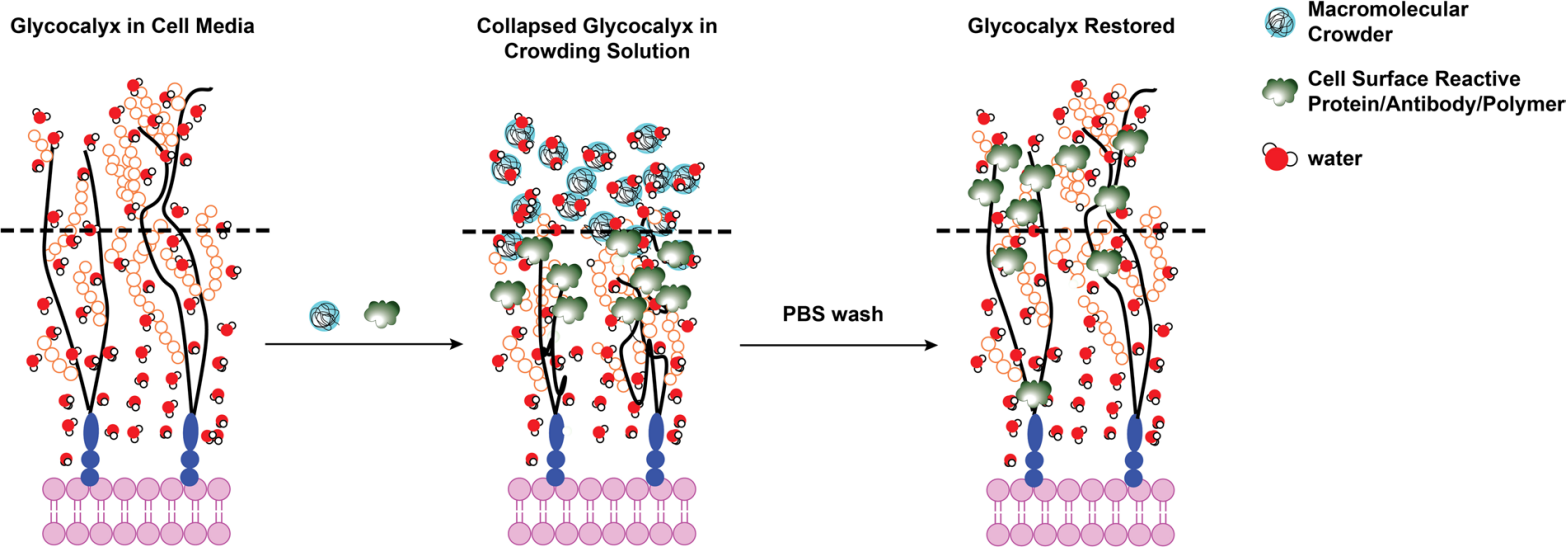
Proposed mechanism for the reversible, oncotically driven collapse of the glycocalyx using inert macromolecular crowders and the preferential grafting or binding of macromolecular binders on the outer surface of the cell surface glycocalyx.

To probe our hypothesis, we imaged the glycocalyx of live HUVECs in the presence and absence of macromolecular crowder using confocal microscopy. The glycocalyx was stained with WGA-633 lectin, a fluorescent marker which binds to sialic acid and *N*-acetylglucosaminyl residues on the proteoglycan structures^49,50^. Following WGA staining with an equal number of WGA proteins (Fig. 5S), the cells were imaged using the similar parameters described in Fig. 3 in order to isolate changes in glycocalyx dimension from overall changes in cell morphology. As shown in Fig. 4a, the height of the glycocalyx significantly decreased in presence of crowder (230 mg/ml) in comparison to non-crowded conditions. The corresponding density (Fig. 4b) of the labelled lectin also increased in the crowding condition, supporting a collapse of the glycocalyx structure. To our delight, after removing the crowding solution, the overall thickness of the glycocalyx returned to its original dimensions (Fig. 4a). Further assays under these conditions also showed no impact on glycocalyx shedding or overall cellular toxicity (Figs 6S & 7S). These results also illustrate the gentle and reversible utility of the current glycocalyx modification approach.

To visualize glycocalyx dehydration, we incubated the HUVEC monolayers in a 1.24 µM solution of 40 kDa FITC-labelled dextran. FITC-dextran has long been used to visualize the movement of molecules within the glycocalyx, as the molecular weight is below the cut-off of the meshwork and it has no significant binding affinity to the glycocalyx structure^51^. Upon the addition of crowding solution, a significant increase in the concentration of the dextran molecules was observed (Fig. 4c), an effect synonymous with fluid removal from the glycocalyx meshwork. It should be noted that transient shrinking of the cell cross-section was observed in the presence of crowder as seen in Fig. 7S. This phenomenon often occurs when cells are placed in cold conditions but may also be due to changes in the osmolarity of the added crowding solution^52,53^. Changes in osmolarity may also contribute to glycocalyx dehydration however, as confirmed by previous work done by McGee and co-workers^54^ it is the oncotic effect of added macromolecular crowders that mediate water transport out of the glycocalyx. Since we used cells that have been cultured for an extended periods of time (21 days) to generate thicker glycocalyxes, the model used by McGee and co-workers (e.g. explants) is more appropriate in the present case. With this, we conclude that the oncotic gradient formed by the introduction of an inert crowder is the main driver of glycocalyx dehydration and subsequent collapse.

We then attempted the utility of this methodology in controlling cell surface binding with non-polymer based macromolecular cell surface markers WGA and a CD138 (syndecan-1) binding antibody. Both WGA and anti-CD138 antibody markers bind to cell surface carbohydrates moieties which play an essential role in endothelial function such as the binding of growth factors, platelets and leukocytes^55,56^. Selectivity for these markers in the *z*-dimension may have use in more acute control over modulating endothelial function, or probing the role of structures on the outer cell surface glycocalyx. As seen in Fig. 4d, redistribution of molecules from the inner (Q1/Q2) to outer surface (Q3/Q4) in crowded conditions was seen for all targets. This finding illustrates the ubiquity of the technique, in that employing macromolecular crowders to redirect cell surface modification can be used for the covalent attachment of polymers and for the binding of fluorescent proteins and antibody markers to the cell surface.

### Enhanced immunocamouflage by crowding-assisted cell-surface engineering

As the location of polymer grafts influences the surface properties of the cells (Fig. 2), we sought to explore some of the biological implications of redirecting graft location in the *z*-direction on cell surface glycocalyx. Using similar HPG-SS grafting conditions as in Fig. 2, we measured the accessibility of antibodies to membrane antigens on polymer grafted RBCs. It has been shown previously that grafting voluminous polymers to the cell surface camouflages antigens embedded in the underlying cell membrane which dictate blood type^12,57^. The steric exclusion driven camouflaging effect depends on the nature of the protective shield on the cell surface. We anticipated that a polymer shield weighted toward the exterior of the glycocalyx would produce greater protection (or camouflage) of cell-surface antigens compared to randomly distributed polymer grafts. As demonstrated in the flow cytometry data summarized in Fig. 5, the accessibility to Rhesus D (R_h_D) and CD47 proteins on the cell surface was significantly decreased in crowding-assisted polymer grafting in comparison to non-crowded conditions. Grafting of 20 kDa HPGs (~9.5 × 10^5^ molecules per cell) in crowded conditions gave ~2-fold greater protection to R_h_D antigens, compared to cells modified with the same number of HPG molecules in the absence of crowder (Fig. 5a). Similarly, the camouflage of CD47 on the cell surface increased when modified cells were prepared in crowded conditions (Fig. 5b). This experiment deviates from the CD-138 binding measured in Fig. 4d as antibody binding is assessed only after crowder has been completely removed. As such, the enhancing effect of crowder assisted camouflage is a result of using transient glycocalyx dehydration to force the formation of a polymer shield that concentrated at the apical region of the glycocalyx (Fig. 5c).

The decrease in enhancement in camouflage with 60 kDa HPG in crowded condition is possibly due to its comparatively larger size which may impede its diffusion into the glycocalyx meshwork. A more exhaustive analysis of 10 other RBC surface antigens also showed improved camouflage in crowded conditions (Table S2). These results clearly support that redistributing HPG towards the exterior of the glycocalyx enhances the desired impact of cell surface engineering with macromolecular agents. Though the re-distribution is small, the biological impact is significant.

## Conclusions

We have presented a ubiquitous, simple, inexpensive and reversible tool which enables controlled modification of the glycocalyx normal to the plane of the membrane in the longitudinal (z) direction, a parameter which has yet to be controlled using existing methods. It was shown that the introduction of macromolecular crowders caused reversible changes in the glycocalyx architecture likely due to oncotic collapse. This collapse sequesters accessibility to the basal region of the glycocalyx, and subsequently enhances modification on the outermost region (Fig. 6).

While HPG was the macromolecular agent used in the current study, in theory other more readily available hydrophilic polymers previously demonstrated as useful in macromolecular crowding (i.e. dextran, ficoll, PEG) may be used to the same effect^31^. This technique was further implicated as applicable to an array of macromolecular targets for the cell surface as shown in protein and antibody binding. We demonstrated that redistributing macromolecular agents to the outermost surface of the glycocalyx correspondingly enhances the intended biological effect of the modification, in our case immunocamouflage of RBC antigens. As the outermost surface of the glycocalyx is the first contact point for cellular response to exogenous effectors, this enhancement is expected to improve the efficacy of other macromolecular therapeutics targeted to the cell surface while minimizing the amount of material used. This new tool for cell surface engineering may be used as an enhancement to established, site-specific engineering approaches intended to tailor cellular behavior. We also envision that this technique could be used to study the relationship between the position of cell surface carbohydrates along the longitudinal axis and their role in cell function.

## Materials and Methods

The following is a sampling of the studies performed which describes the materials and methods for key experiments. Whole blood was collected from healthy donors into a citrate vacutainer tubes. Procedures involving human subjects have been approved by the Institutional Review Board (IRB) at the University of British Columbia and all methods were performed in accordance with the relevant guidelines and regulations of the IRB including informed consent from all human participants in this study. Detailed materials and methods can be found in the supporting information.

### HPG grafting to RBC surfaces (non-crowded)

Immediately after preparation, 100 mg of 20 kDa succinimidyl succinate activated (HPG SS) was dissolved in 120 µL of phosphate buffered saline (pH 8.0). Concentrated RBCs were added to a phosphate buffered saline solution (pH 8.0) followed by freshly prepared HPG-SS stock solution. For example, to obtain 1 mM of 20 kDa HPG-SS polymer graft solution, 40 µL of polymer solution was added to RBC suspension (20% Hematocrit) to a final volume of 600 µL. The mixture was vortexed gently and placed on an orbital shaker at room temperature for 1 h. Cells were washed 2 times with PBS buffer, once with saline, re-suspended in saline to 20% Hematocrit and used for further analysis.

### HPG grafting to RBC surfaces (crowding treatment)

Immediately after preparation, 100 mg of 20 kDa succinimidyl succinate activated (HPG SS) was dissolved in 120 µL of phosphate buffered saline (pH 8.0). Concentrated RBCs were added to a solution of macromolecular crowder dissolved in phosphate buffered saline solution (pH 8.0) followed by freshly prepared HPG-SS stock solution. For example, to obtain 1 mM of 20 kDa HPG-SS polymer graft solution, 40 µL of HPG20K polymer solution was added to RBC suspension (20% Hematocrit) to a final volume of 600 µL and final macromolecualr crowder concentration of 230 mg/mL. The mixture was vortexed gently and placed on an orbital shaker at room temperature for 1 h. Cells were washed 2 times with PBS buffer and 1 time with saline and suspended in saline to 20% Hematocrit.

### Aqueous two-phase partitioning measurements

The aqueous two phase partitioning system was made of 4% (w/w) PEG 8 kDa, 5% (w/w) dextran 500 kDa, supplemented with 150 mM of NaCl, 8.06 mM of dibasic potassium phosphate, and 1.94 mM of monobasic potassium phosphate. In 1 mL of dextran/PEG two phase aqueous partitioning solution, 5 μL of packed RBCs were added. The mixture was vortexed gently and placed on the benchtop for 10 min at room temperature. Based on surface properties of RBCs, the level of functionalization, RBCs were positioned in the lower dextran phase, the upper PEG phase or in both phases.

### Alexa Fluor 633 tagged HPG-SS labelling of HUVEC monolayers (non-crowded)

For polymer labeling of the cell surface, HUVECs were washed three times with DPBS and treated with a step-wise addition of DPBS followed by Alexa Fluor-633 labeled HPG-SS (20 kDa) to a final concentration of 0.7 mM HPG-SS. The solution was gently mixed for 30 seconds and the cells were incubated for 1 hour at 4 °C under static conditions. Following incubation, monolayers were washed twice with room temperature DPBS and stained with Hoescht 3388 (1 µg/mL) and CellMask green plasma membrane stain (1:1000 × dilution) for 15 minutes at 37 °C. Following staining, the cells were washed three times with DPBS and immersed in EGM-2 (phenol free, 15 mM HEPES) for live cell imaging. All experiments were repeated three times (3 independent experiments) and the results pooled.

### Alexa Fluor 633 tagged HPG-SS labelling of HUVEC monolayers (crowding treatment)

For polymer labeling of the cell surface, HUVECs were washed three times with DPBS and treated with a step-wise addition of crowding solution (30 kDa HPG (unmodified) in DPBS, sterilized through filtration using a 0.22 µm membrane) followed by Alexa Fluor-633 labeled HPG-SS (20 kDa) to a final concentration of 230 mg/mL crowding solution and 0.5 mM HPG-SS. The cells were incubated for 10 minutes in crowding solution at ambient temperature before the addition of HPG-SS. The solution was gently mixed for 30 seconds and the cells were incubated for 1 hour at 4 °C under static conditions. Following incubation, monolayers were washed twice with room temperature DPBS and stained with Hoescht 3388 (1 µg/mL) and CellMask green plasma membrane stain (1:1000 × dilution) for 15 minutes at 37 °C. Following staining, the cells were washed three times with DPBS and immersed in EGM-2 (phenol free, 15 mM HEPES) for live cell imaging. All experiments were repeated three times (3 independent experiments) and the results pooled.

### Visualization of the glycocalyx with FITC labelled dextran

HUVECs were washed three times with DPBS and stained with Hoescht 3388 (1 µg/mL) and CellMask^TM^ deep red plasma membrane stain (1:1000 × dilution) for 25 minutes at ambient temperatures. Following staining, the cells were washed three times with DPBS and treated with a step-wise addition of crowding solution (30 kDa HPG (non-modified) in EGM-2, sterilized through filtration using a 0.22 µm membrane) or an equal volume of EGM-2 (phenol free, 15 mM HEPES) followed by FITC-Dextran (Sigma, FD40S) to a final concentration of 1.24 µM FITC Dextan and 230 mg/mL crowding solution. Cells were immediately visualized using confocal microscopy. To account for stepwise photobleaching, the average slice intensity within the glycocalyx (Fig. 8S, dashed border) was normalized to the intensity of the FITC signal outside the glycocalyx (Fig. 9S). HUVEC cell membranes were labeled with CellMask^TM^ deep red plasma membrane stain to mark the base of the glycocalyx structure. All experiments were repeated three times (3 independent experiments) and the results pooled.

### CD 47 cell surface protein analysis

RBCs (10 µL, 3.3% Hematocrit) were mixed and 80 ul PBS buffer and with Phycoerythin (PE) mouse anti human CD47 (10 µL, 2/10 dilution, 0.5% BSA) from BD Biosciences, Canada. The mixture was incubated at RT for 30 min in dark. The RBCs suspension was washed one time with 1 mL of PBS buffer with 0.5% BSA and re-suspended into 0.5 mL. The suspension was passed through a 25 G5/8 needle to minimize cell clumping and analyzed using a BD FACS Canto II flow cytometer acquiring 10000 events.

### Rhesus D antigen analysis

FITC-labeled mouse anti human Rhesus D (13 µL), obtained from Quotient Biodiagnostics, PA, USA was added to the RBC suspension (37 uL, 1.3% hematocrit), mixed carefully and incubated at RT for 30 min in the dark. RBCs were washed one time with PBS buffer pH 7.4. The supernatant was removed and then RBCs were dispersed in PBS buffer (0.5 ml). The suspension was analyzed using a BD FACS Canto II flow cytometer acquiring 10000 events using medium flow.

### Statistical analysis

All data are presented as a mean ± the standard deviation unless otherwise mentioned. Where indicated, 2-tailed t-tests analysis with Welch’s correction was performed using Graphpad Prism 7.0. Paired comparisons were significant when *p* < 0.05 (*), *p* < 0.01 (**) and *p* < 0.001 (***). All experiments were repeated three times (3 independent experiments) and the results pooled unless otherwise noted.

### Data availability

All data generated or analysed during this study are included in this published article (and its supplementary information file).

## Electronic supplementary material

Supporting Information
