# Supplementary material for: Oncotically Driven Control over Glycocalyx Dimension for Cell Surface Engineering and Protein Binding in the Longitudinal Direction
Source: Sci Rep. 2018 May 15;8:7581. doi: 10.1038/s41598-018-25870-2 (PMC5954099; doi:10.1038/s41598-018-25870-2)
Supplement: Supplementary file 1 — Supporting Information [file 41598_2018_25870_MOESM1_ESM.docx]

**Oncotically Driven Control over Glycocalyx Dimension for Cell Surface Engineering and Protein Binding in the Longitudinal Direction**

Erika M.J. Siren,^1,2^ Rafi Chapanian,^1,3^ Iren Constantinescu,^1,3^ Donald E. Brooks,^1,2,3^ and Jayachandran N. Kizhakkedathu^1,2,3,*^

^1^Centre for Blood Research, ^2^Department of Chemistry, ^3^Department of Pathology and Laboratory Medicine, Life Sciences Centre, University of British Columbia, Vancouver, BC, Canada, V6T 1Z3

**Supplementary Information**

**Reagents and Materials.**

**Chemical and Biochemical Materials.** Chemicals used in polymer synthesis and modification include trimethylolpropane (TMP), obtained from Fluka (ON, Canada); glycidol, potassium methylate, 1,4-dioxane, succinic anhydride, 4-dimethylaminopyridine, N,N′-diisopropylcarbodiimide, N-hydroxysuccinimide, and Amberlite IR120 H-ion-exchange resin from Sigma Aldrich (ON, Canada), and Alexa Flour-633® hydrazide from Thermo Scientific. Cellulose ester dialysis membranes with a MWCO of 1 kDa were obtained from Spectra/PorBiotech (CA, USA). Glycidol was distilled and dried over 4 Å molecular sieves prior to use. Solvents, anhydrous pyridine, anhydrous dimethyl formamide, anhydrous N-methyl pyrrolidine, methanol, and acetone were purchased from Sigma Aldrich and used without purification. Tritium labeled methyl iodide was purchased from American Radiolabeled Chemicals Inc. (St. Louis, USA). FITC-labeled mouse anti human Rhesus D was purchased from Quotient Biodiagnostics (PA, USA), and Phycoerythin (PE) mouse anti human CD47 from BD Biosciences (NJ, USA).

**Cell Culture Materials**. All cell culture-related medium and supplements (Trypsin-EDTA, Dulbeccos phosphate-buffer saline (DPBS), HT fetal bovine serum (FBS), penicillin/streptomycin (P/S), and Endothelial Growth Medium (EGM-2 ) were received from Life Technologies Inc. unless otherwise specified. Primary Human Umbilical Vein Endothelial cells (HUVECs) were purchased from American Type Culture Collection (ATCC; Manassas, VA) and used up to a passage number of seven.

**Techniques.** Absolute molecular weights of the polymers were determined by Gel Permeation Chromatography (GPC) on a Waters 2695 separation module fitted with a DAWN EOS multiangle laser light scattering (MALLS) detector coupled with Optilab DSP refractive index detector, both from Wyatt Technology. GPC analysis in aqueous conditions was performed using Waters ultrahydrogel columns (guard, linear and 120) and 0.1 N NaNO_3_ at pH 8.5 (10 mM phosphate buffer) as the mobile phase. Waters styragel columns (guard, HR3 and HR4) were used for the GPC analysis in chloroform. The dn/dc value used for polyglycerols (determined at λ= 690 nm 0.1 N NaNO_3_) was 0.12. ^1^H NMR spectra were recorded on a Bruker Advance 300 MHz NMR spectrometer. D_2_O (Cambridge Isotope Laboratories, Andover MA) was used as solvent, with the relevant solvent peak as reference. UV-Vis spectra were recorded on Varian Cary Eclipse spectrophotometer. All confocal images were acquired using an inverted Zeiss Axiovert 200M spinning disk confocal microscope (Oberkochen, Germany). Unmodified HPG (30 kDa) was used as a macromolecular crowder for the experiments.^1^ The concentration of macromolecular stock solutions was measured using a TA Instruments Q500 Thermogravimetric Analyzer (New Castle, USA) over a temperature range of 25 °C-300 °C and a ramping rate of 10 °C/min

**Polymer Synthesis and Functionalization**

Hyperbranched polyglycerol (HPG) was synthesized by the ring opening multi-branching anionic polymerization of glycidol ^2-5^. Briefly, trimethylolpropane was partially deprotonated (~ 10%), using potassium methylate, methanol was removed under vacuum, and glycidol was added drop wise over several hours at ~ 100 °C using a syringe pump, the mixture was stirred for an additional 8 hrs. The polymer was dissolved in methanol at a ratio of (2:1) (methanol/polymer) and treated by passing twice through Amberlite IR120 H-ion-exchange resin column. The polymer was purified by dialyzing against water for 3 days using a 3.5-5 kDa MWCO cellulose membrane and freeze drying. Purified polymer (*M_n_* -30,000 g/mole and M_w_/M_n_- 1.28) was fractionated by precipitating its methanol solution into acetone to obtain polymers with varying molecular weights (20, 30 kDa) and narrow M_w_/M_n_ (~ 1.1). Obtained polymers were dried and dialyzed in water to remove any remaining organic solvents and submitted for structural analysis (Figure 2S)

^1^H NMR (300 MHZ, D_2_O, ppm) δ: 3.61-3.98 (m, HPG backbone –CH2O-, -CHO-),

**Carboxyl Group Functionalization of HPG**

To prepare the polymer for modification, HPG (Mn 20 kDa, 954 mg, 0.047 mmol,) was dried *in vacuo* at 90 C overnight and subsequently dissolved in anhydrous pyridine (19 mL). Ten molar equivalents of succinic anhydride (470 mg, 0.470 mmol) was added to the solution followed by the slow addition of dimethylaminopyridine (46.09 mg, 0.376 mmol) which was then allowed to react under Argon at room temperature overnight. Upon reaction completion, the polymer was precipitated from the reaction medium with acetone and isolated via centrifugation (14,000 *g,* 4 °C). The polymer was then redissolved in distilled water and dialyzed against 1K MWCO tubing, lyophilized and subjected to structural analysis (Figure 3S).

^1^H NMR (300 MHZ, D_2_O, ppm) δ: 3.61-3.98 (m, HPG backbone –CH2O-, -CHO-), 2.59 (s, COCH2CH2COOH), 2.45 2.59 (s, COCH2CH2COOH).

**Preparation of Hyperbranched Polyglycerol Succinimidyl Succinate (HPG-SS)**

Carboxyl group functionalized HPG (226 mg, 0.0113 mmol) and N-hydroxysuccinimide (7.97 mg, 0.0678 mmol) was re-dissolved in anhydrous DMF (9 mL) followed by the addition of N,N′-Diisopropylcarbodiimide (8.75 mg, 0.0678 mmol). The solution was left to stir overnight at room temperature under argon atmosphere. Upon reaction completion, the solution was precipitated in acetone (30 mL), isolated by centrifugation (14,000 *g,* 4 °C), and dried for 10 minutes under reduced pressure. Once isolated, HPG-SS was immediately used for derivatization with the cells. The polymer was dissolved in a PBS stock solution prior to grafting to the cells. Modification was verified via UV measurement following base mediated ester hydrolysis (λ_max(NHS-OH)_ = 260 nm)

**Radio-labeling of HPGs (^3^H-HPGs)**

A detailed procedure for HPG radiolabeling has been reported in previous work.^2^ Briefly, dried HPG was dissolved in N-methylpyrolidone, deprotonated partially (~ 10%) and reacted with tritium labeled methyl iodide (C^3^H_3_I) to obtain less than 1 % of HPG hydroxyl groups methylated. The reaction was stopped by a slow addition of water. Unreacted reagents were removed by dialysis against water with 1 MWCO dialysis tubing. The specific activity of the polymer was determined from the volume of the polymer solution, polymer weight, and radioactivity. The acid functionalization and N-hydroxysuccinimide (NHS) activation of ^3^H-HPGs were performed using steps similar to those used with cold HPGs. Prior to radioactivity measurements, RBCs were digested according to our previous procedure.^2^ RBCs were digested to homogenize the colloidal cell solutions for scintillation counting to measure the radioactivity accurately. The number of HPG molecules grafted per RBC was quantified from the radioactivity of a known volume of packed-RBCs, the specific activity of the polymer, and the density of RBC.^6^

**Alexa Fluor 633/BODIPY FL labeling of HPGs:**

Fluorescent labeling of HPGs was performed by functionalizing 20 mol % of HPGs with Alexa Fluor® 633 Hydrazide (Thermo Fisher Scientific) through reductive amination. Aldehyde groups were generated on HPG though the oxidation of 1,2 diol groups on the polymer scaffold using sodium periodate (NaIO_4_). HPG (140 mg, 0.0071 mmol) was dissolved in PBS (1.0 M, pH 7.0) followed by the addition of NaIO_4_ (0.37 mg, 0.0017 mmol). The solution was stirred overnight at room temperature protected from light. Following periodate oxidation the solution was quenched with glycerol to a final concentration of 20 mM. Alexa Fluor® 633 Hydrazide (0.0012 mmol) dissolved in DMSO was added to the solution followed by an aniline stock solution (1 M in DMSO) to a final aniline concentration of 10 mM. The mixture was left for 4 hours at room temperature protected from light. Following reaction completion, the reducing agent NaBH_4_ (0.45 mg, 0.012 mmol) was added to the solution and left to stir overnight at room temperature protected from light. Following reduction, an excess of glycine was added to a final concentration of 10 mM in order to quench any remaining aldehyde groups and stirred for another hour. The conjugate solution was lyophilized, dissolved in methanol and precipitated in cold ether 3 times. The precipitate was then dissolved in water and purified through dialysis (3.5 MWCO) until a negative silver nitrate test was obtained. For BODIPY labeling of HPG, the procedure was repeated using BODIPY-FL hydrazide (Thermo Fisher Scientific).

**Cell Based Assays**

**RBC isolation.** Whole blood was collected from healthy donors into a citrate vacutainer tubes. Procedures involving human subjects have been approved by the Institutional Review Board (IRB) at the University of British Columbia and all methods were performed in accordance with the relevant guidelines and regulations of the IRB including informed consent from all human participants in this study. For RBC isolation, tubes were centrifuged at 1000 x g for 4 min. The supernatants along with buffy coat were removed using a Pasteur pipette. RBCs were washed three times with saline and suspended in PBS buffer (pH 8.0).

**Electrophoretic mobility analysis.** The electrophoretic mobility experiments were conducted using an instrument made for G.V.F. Seaman based on the Rank Brothers Mark I Electrophoresis apparatus (Rank Brothers, Bottisham, UK). A drop of 20% Hematocrit of RBC was diluted in 10 ml saline and the velocity of individual cells located in the stationary region was measured at a constant potential. The electrophoretic mobility was calculated from the following equation:

$EM=\frac{v\times L}{V}$

Where, *v* is the measured velocity of individual cells, *V* is the applied voltage (40.5 V), and *L* is the effective electrical length of the chamber. At least 10 individual cells were selected randomly and their velocity was measured, results were presented as the mean ± standard deviation.

The electrophoretic mobility of RBCs and HPG-grafted RBCs was measured at different NaCl concentrations in the range 0.02 – 0.154 mM. To maintain the isotonicity of RBCs, solutions were supplemented with anhydrous dextrose in cases where NaCl concentrations were below 0.154 mM. The viscosity of solutions was measured using Cannon-Manning 50, A 412 semi-viscometer.

To interpret the electrophoretic mobility of HPG-grafted RBCs, numerical values for the following parameters were required^7,8^: the surface charge density (σ), the thickness of glycocalyx (β), the Stokes effective mean radius of the segment of glycocalyx (a), and total mass on the surface.

We assumed that grafting of neutral HPGs does not introduce changes in cell surface charges and charge distributions. We also assumed that the thickness of the native glycocalyx structure remained unchanged as a result of grafting of HPGs. A depletion of the whole cell volume was observed in crowded conditions however, this effect was transient with cell size returning to normal values when removed from crowding solution. Thus we used values of σ (1.065 x 10^4^ esu) and β (7.8 nm) that have been reported for naïve RBCs^7,8^.

The Stokes mean segment radius of glycocalyx, was determined by fitting the calculated electrophoretic mobility values to experimental data in a manner similar to Levine S. *et al.*^7^.

The weight of glycocalyx in HPG-grafted RBCs was calculated to be the mass of glycocalyx in naïve cells (~ 1.18 x 10^-13^ g)^7^ plus the weight of HPG grafts that were measured using the tritium labeled ^3^H-HPG-RBCs.

In the case of RBCs grafted with HPGs, we assumed that macromolecules are distributed randomly within the glycocalyx, considering the small hydrodynamic sizes of HPG (20K) relative to glycocalyx (R_h_ = 3.0 nm).

The model developed by Sharp and Brooks^8^, allows varying the locations of bound polymers within the glycocalyx. The impact of HPG graft location within glycocalyx on electrophoretic mobility was investigated. We further fitted the calculated values of electrophoretic mobility, using determined parameters, to experimental data of HPG-grafted RBCs at different ionic strengths to investigate whether the extracted Stokes effective mean radius of the segment of glycocalyx (a) at 0.154 mM saline is applicable at other ionic strengths (Figure 3S).

**Cell Culture**. HUVECs were plated and cultured on IBIDI µ-Slide 8 well chamber slides in EGM-2 Media (2% FBS, 1% P/S). Cells were seeded at a concentration of 10,000 cells/well and cultured for 21 days (37 °C, 5% CO2) with a change of media every two days.^9^

**Microscopy experiments**. All confocal images were acquired using an inverted Zeiss Axiovert 200M spinning disk confocal microscope equipped with a QuantEM 512SC Photometrics camera (512x512 pixels size) and an incubator platform for live-cell imaging. Images were captured in series using a 100X/1.45 Oil Plan-Fluor objective lens coupled to a spherical aberration correction unit. *z*-axis profiles were constructed by stepping the focus in 200 nm increments: A typical *z*-axis profile was carried out over a cell surface starting from the cell membrane (intensity maxima of the membrane stain) through the glycocalyx and into the solution above. 5 different regions of each cell were imaged, with a total of 5 randomly selected cells measured in each experiment. All experiments were repeated three times (3 independent experiments) and the results pooled. Images of live cells were acquired within one hour of staining.

**PE- conjugated Anti- human CD138 staining labelling of HUVEC monolayers (non-crowded).** HUVECs were washed three times with DPBS and treated with a step-wise addition of DPBS followed by PE-conjugated Anti-human CD138 (mouse mAb, clone BA38, Exbio, Vestec, Czech Republic) to a final antibody concentration of 7:100. The solution was gently mixed for 30 seconds and the cells were incubated for 45 minutes at 4 °C under static conditions. Following incubation, monomers were washed twice with room temperature DPBS and stained with Hoescht 3388 (1 µg/mL) and CellMask green plasma membrane stain (1:1000 x dilution) for 15 minutes at room temperature. Following staining, the cells were washed three times with DPBS and immersed in EGM-2 (phenol free, 15 mM HEPES) for live cell imaging. All experiments were repeated three times (3 independent experiments) and the results pooled.

**PE- conjugated Anti- human CD138 staining labelling of HUVEC monolayers (crowded).**

HUVECs were washed three times with DPBS and treated with a step-wise addition of crowding solution (30 kDa HPG (unmodified) in DPBS, sterilized through filtration using a 0.22 µm membrane) followed by Anti-human CD138 (mouse mAb, clone BA38, Exbio, Vestec, Czech Republic) to a final concentration of 230 mg/mL crowding solution and an antibody concentration of 1:20. The cells were incubated for 10 minutes in crowding solution at ambient temperature before the addition of antibody. The solution was gently mixed for 30 seconds and the cells were incubated for 45 minutes at 4 °C under static conditions. Following incubation, monomers were washed twice with room temperature DPBS and stained with Hoescht 3388 (1 µg/mL) and CellMask green plasma membrane stain (1:1000 x dilution) for 15 minutes at room temperature. Following staining, the cells were washed three times with DPBS and immersed in EGM-2 (phenol free, 15 mM HEPES) for live cell imaging. All experiments were repeated three times (3 independent experiments) and the results pooled.

**Alcian blue dye binding assay for quantification of sulfated glycosoamino glycans (GAGs)**

HUVECs were plated and cultured on 6 well multi-well plates in EGM-2 Media (2% FBS, 1% P/S). Cells were seeded at a concentration of 60,000 cells/well and cultured for 21 days (37 °C, 5% CO2) with a change of media every two days. For the TNF-alpha control, cells were subjected to 100 U TNF-alpha (abcam, Cambridge, United Kingdom) for 16 hours. Cell media (100 uL) was treated with alcian blue, isolated according to literature procedures and quantified via absorbance in a 96 well plate at 490 nm.^10^ Absorbance was then read at 490 nm. The ionic interaction between the cationic dye (Alcian blue) and the negatively charged GAG is proportional to the number of negative charges present. The concentration of sulfated GAG in the samples was quantified using a Chondroitin Sulfate (Sigma) standard curve (0.02–0.313 mg/ml). Results were normalized to total cell count via trypan blue exclusion staining (Figure 6S). This experiment was completed in triplicate.

**Cell viability assay for HUVECs in the presence of macromolecular crowding solution**

HUVECs seeded in a 96 well plate (10,000 cells/well) and cultured for 21 days were subjected to crowding and non-crowding conditions. At the end of the exposure (1 hour, 4 C), the supernatants were removed and aliquots of 100 µl were kept for LDH testing. Cell viability was assessed by the measurements of lactate dehydrogenase (LDH) enzyme in the supernatant of the cell culture (Figure 7S). Leakage of this enzyme from the cytoplasm into the supernatant is characteristic of membrane damage. The assay was performed using the LDH-Cytotoxicity Colorimetric Assay Kit (Biovision Incorporated, San Francisco, USA) according to the manufacturer’s protocol. The LDH concentration in 100 µl of the cell culture supernatant was determined at a wavelength of 492 nm. Cells treated with 1 % (w/v) Triton X-100 (10 minutes, room temperature) served as reference for the maximum possible LDH release (100%, high control). The relative LDH release of a given sample is then defined as the ratio of LDH measured in the supernatant of the sample and the high control value with LDH values under 20% regarded as a nontoxic effect level. All experiments were repeated twice times (2 independent experiments) and the results pooled.

**Enzyme-mediated glycocalyx removal from Endothelial Cell Surface.**  Washed HUVECs (14 days old culutre) were treated with 0.5 mM HPG-SS (20 kDa, 10% BODIPY FL) in PBS. The solution was gently mixed for 30 seconds and the cells were incubated for 1 hour at 4 °C under static conditions. Following incubation, monolayers were washed twice with room temperature DPBS and stained with Alexa-633 conjugated WGA (10 µg/mL) in PBS for 20 minutes at ambient temperature. The doubly labeled cells were then incubated with serum starved cell media containing 0.1 mM H_2_O_2_ and 1 nM epinephrine for 1 hour at 37 °C according to previous procedures.^11^ The stimulation of the cells with reactive oxygen species (H_2_O_2_) and catecholamines (epinephrine) has been demonstrated to upregulate the expression of glycocalyx degrading extracellular proteases including human matrix metalloprotease’s (MMP). Cells were washed, trypsinized and immediately subjected to flow cytometry. Controls either a) without any label or b) singly labeled, no enzyme treatment were used to set gates for 100% labeled cell populations.

**Enzyme-mediated glycocalyx removal from RBC Surface.**  Washed RBCs and were added to PBS to a final concentration of 10% hematocrit treated with 0.5 mM HPG-SS (20 kDa, 10% BODIPY FL). The solution was gently mixed for 30 seconds and the cells were gently rocked for 1 hour under ambient conditions. Following incubation, monolayers were washed twice with room temperature DPBS and stained with Alexa-633 conjugated WGA (10 µg/mL) in PBS for 20 minutes at ambient temperature. The doubly labeled cells were then incubated with APMA activated human MMP 2,7 and 9 (120 nM for all enzymes) for 1 hour at 37 °C according to previous procedures.^12^ Cells were washed and immediately subjected to flow cytometry. Controls either a) without any label or b) singly labeled, no enzyme treatment were used to set gates for 100% labeled cell populations.

**Standard hospital phenotyping analysis for RBC minor antigens.** The protection of several minor antigens on modified RBCs under crowded and non-crowded conditions including Rhesus (D, C, c, E, e), Kell (K and k), Duffy (Fy^a^ and Fy^b^) Kidd (JK^a^ and JK^b^) Lewis (Le^a^ and Le^b^) MNSs (M, N, S and s), P (P_1_), C_w_ antigens were conducted using standard hospital phenotyping techniques at St. Paul’s hospital in Vancouver, BC. The test principle in phenotyping for minor antigens lies in the reaction of a particular minor antigen on the surface RBCs with the corresponding monoclonal antibody that results in RBC agglutination. Briefly, to subtype for rhesus antigens D, C, c, E, and e, RBCs are suspended at a concentration 3-5% in isotonic saline solution, 1 drop of corresponding antibody reagent (Bio-Rad, ON, Canada) are mixed with 1 drop of suspended RBCs, the mixture is incubated for 5 min at room temperature (22 °C). The mixture is centrifuged for 2 min at 175 x g, and the product is dislodged gently to evaluate the extent of agglutination. Minor antigen expression is evaluated on a score from +4 to 0. A score of +4 is given when a single agglutinate is formed, +3 when some large agglutinates are formed, +2 for medium size agglutinates with no free cells, +1 for few macroscopically detectable agglutinates within a homogenous RBC suspension, and 0 for homogenous RBC suspension with no detectable agglutinates. A score of 0 indicates to the absence of a particular antigen in the control RBC, whereas, a score +4 indicates a strong expression of a particular antigen on the surface of RBCs.

**Statistical analysis.** All data are presented as a mean ± the standard deviation unless otherwise mentioned. Where indicated, 2-tailed t-tests analysis with Welch’s correction was performed using Graphpad Prism 7.0. Paired comparisons were significant when *p* < 0.05 (*), *p* < 0.01 (**) and *p* < 0.001 (***). All experiments were repeated three times (3 independent experiments) and the results pooled unless otherwise noted.

**
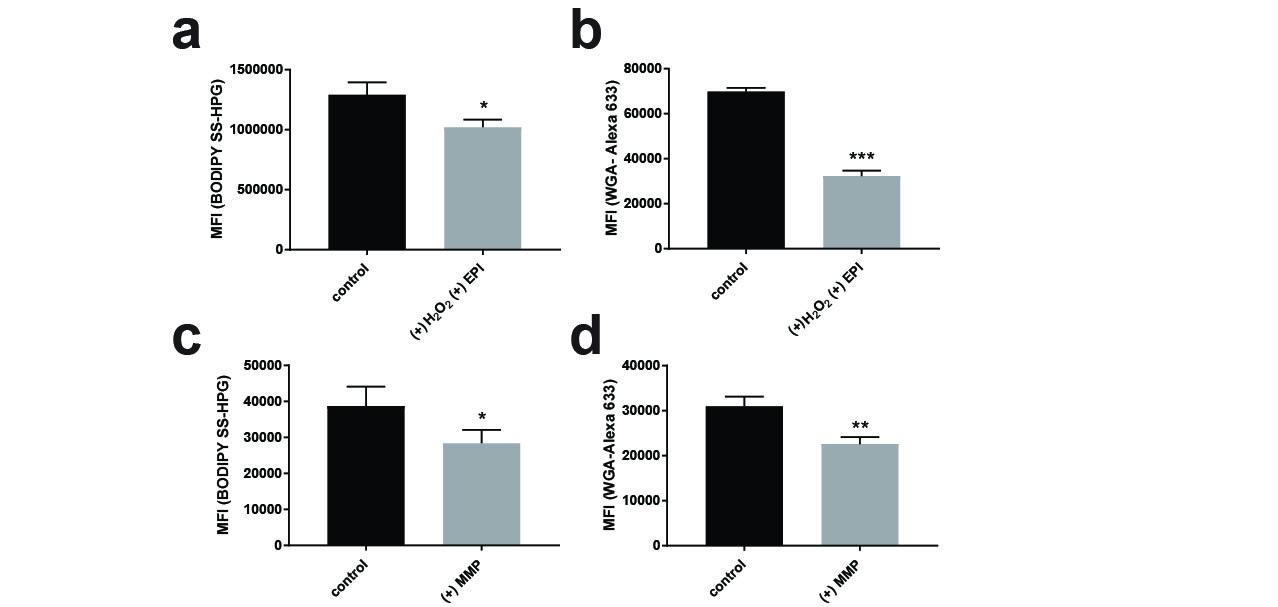
**

**Figure 1S.** Extent of HPG grafting to glycocalyx. HUVEC and RBC cells were incubated with 0.5 mM SS-HPG (% BODIPY FL) in PBS, labeled with glycocalyx marker WGA-Alexa 633, and subjected to analysis by flow cytometry before and after enzyme treatment. Labeled HUVEC monolayers were treated with 0.1 mM H_2_O_2_ and 1 nM epinephrine (EPI) to stimulate glycocalyx shedding and the extent of polymer graft (a) and glycocalyx (b) loss were measured. Labeled RBCs were treated with 120 nM of activated MMP-2,7 and 9 to force glycocalyx shedding and the extent of polymer graft (a) and glycocalyx (b) loss were measured. Enzyme-mediated removal of both glycocalyxes caused a corresponding decrease in surface grafted HPG polymer suggesting that the polymer is grafted preferentially onto glycocalyx (see also Table 1S). MFI- mean fluorescence intensity. Unpaired comparisons using a non-parametric t-test are significant with *p* > 0.05 (*) and *p* > 0.01 (**).

**Table 1S:** Extent of glycocalyx labeling by SS-HPG

| **Cell Type** | **Marker/Channel** | **Decrease in Marker Intensity following Partial Glycocalyx Removal (%)** |
| --- | --- | --- |
| HUVEC | HPG/FITC | 20 ± 4.9 |
|  | WGA/APC | 53 ± 3.3 |
| RBC | HPG/FITC | 26 ± 9.4 |
|  | WGA/APC | 27 ± 4.9 |

** Removal of the entire glycocalyx resulted in significant cell lysis for both cell types. As such, gentle conditions were used to provoke some glycocalyx removal without impacting cell viability.


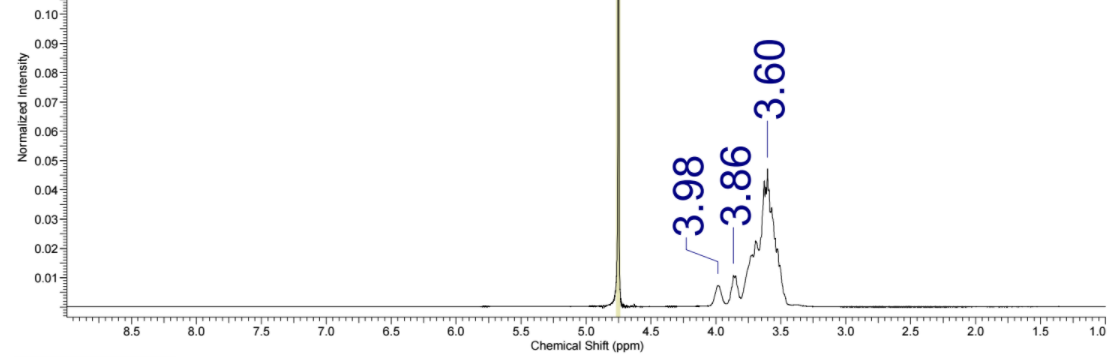


**Figure 2S.** ^1^H NMR of inert, unmodified HPG. ^1^H NMR (300 MHz, D_2_O): δ=3.86-3.59 (m, HPG backbone, 5H)


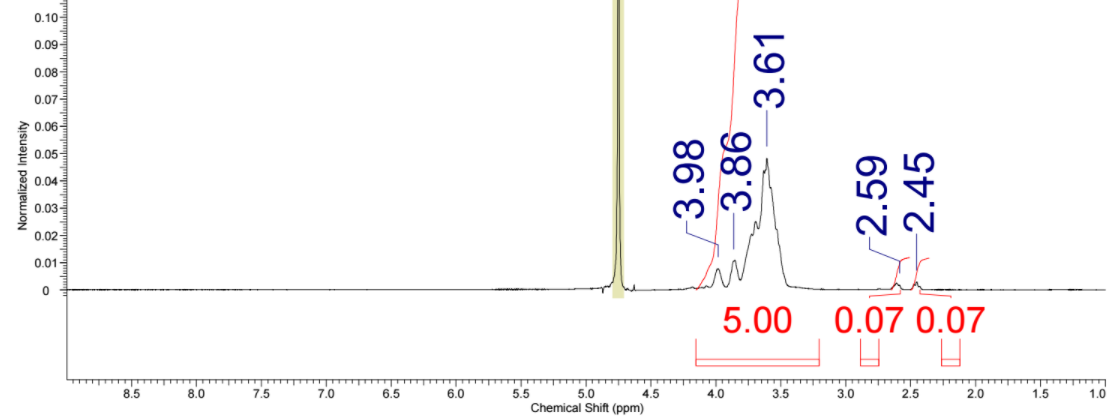


**Figure 3S.** ^1^H NMR of carboxyl modified HPG. ^1^H NMR (300 MHz, D_2_O): δ=3.86-3.59 (m, HPG backbone, 5H), 2.68 (m, 2H), 2.41 (m, 2H)


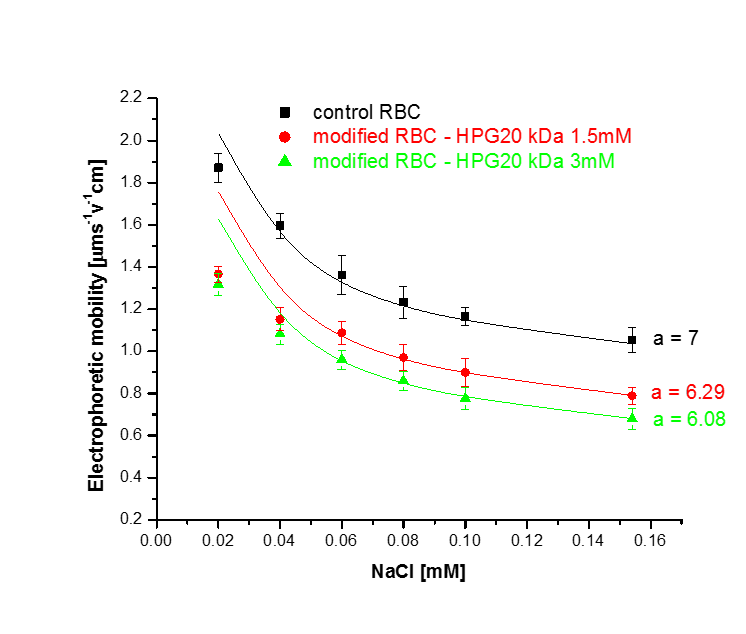


**Figure 4S:** Mathematical simulation used to predict electrophoretic mobility of RBCs at different NaCl concentrations using a mean segment radius of glycocalyx extracted at 0.154 mM.


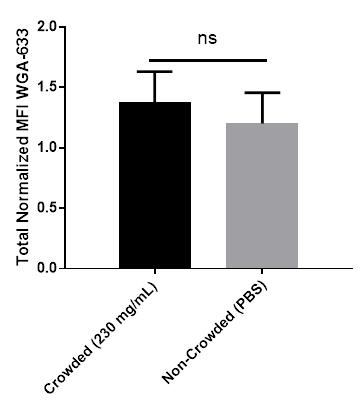


**Figure 5S:**  Total intensity of Alexa Fluor-633 labeled WGA attached to HUVEC glycocalyxes in both crowded (230 mg/mL 30 kDa HPG) and non-crowded conditions. Results show that there is no significant difference between the degree of modification in both conditions when 5 µg/mL and 6.1µg/mL WGA-633 are used for crowded and non-crowded conditions respectively. WGA intensity values were normalized to cell number and are presented as a ratio against Hoescht nuclear stain intensities. The data represents the average of three independent experiments with replicates. Error bars represents standard deviations. Unpaired comparisons using a non-parametric t-test are significant with *p* > 0.05 (ns).


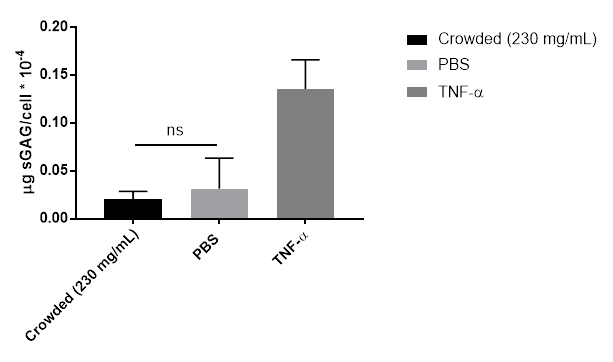


**Figure 6S:** Glycocalyx shedding in the presence of different modification conditions. Polycationic alcian blue dye tightly binds to sulfated glycosaminoglycans (sGAGs) present in cell media. TNF-alpha (100 U/Ml, overnight incubation at 37 °C, 5% CO_2_) has been proven to induce glycocalyx shedding and has been used as a positive control.^13^ Results show that there is no significant difference in glycocalyx shedding in both conditions. Plotted values for µg sGAG in cell media are normalized to cell number as quantified by trypan blue assay. The data represents the average of three independent experiments with replicates. Error bars represents standard deviations. Unpaired comparisons using a non-parametric t-test are significant with *p* > 0.05 (ns).


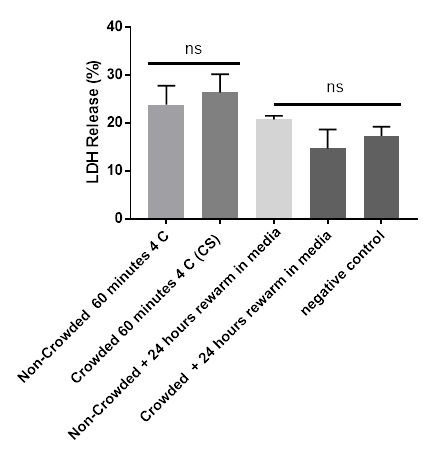


**Figure 7S.** Cell viability of HUVECs subjected to different modification conditions (crowded and non-crowded). LDH release corresponds to damage to the cell membrane, an indicator of poor cell health. All values were normalized to a positive control of an equal number of HUVEC’s subjected to 1% triton-x detergent. The data represents the average of two independent experiments with replicates. Error bars represents standard deviations. Unpaired comparisons using a non-parametric t-test are significant with *p* > 0.05 (ns).


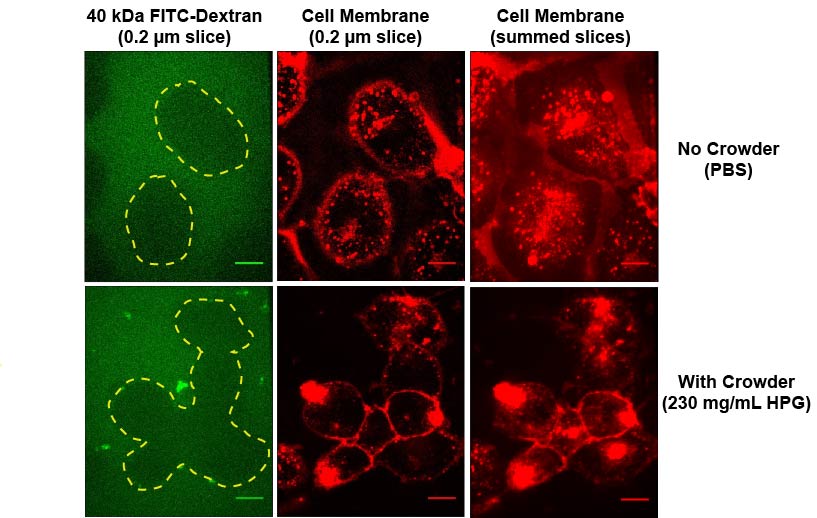


**Figure 8S.** Representative confocal images of HUVECs incubated in 1.24 µM FITC-labeled dextran in crowded and non-crowded conditions. Yellow dashed lines denote border in which glycocalyx begins. HUVEC cell membranes were labeled with CellMaskTM deep red plasma membrane stain to mark the base of the glycocalyx structure. As images were acquired while cells were immersed in crowding solutions, changes in cell morphology due to differences in the osmolarity of the cell media are observed in the summed slices. Scale bar represents 10 µm.

Cell-surface presentation of HS GAGs on ESCs induces FGF2-mediated ERK1/2

activation. A) Representative immunoblots (left) and quantification (right) of ERK1/2

phosphorylation levels in ESCs remodeled with the indicated HS GAGs and stimulated

with FGF2. Phospho-ERK levels were normalized with respect to the total ERK levels for

each condition and compared to those of untreated ESCs. Tubulin was used as a control

for equal protein loading. Data represent the mean  SEM (*P < 0.05) from three

experiments. B) FGFR1-Fc binding to glycan microarrays in the presence (blue) or

absence (black) of FGF2. Data represent the mean  SEM from ten replicate microarray

spot**Materials and Methods:**


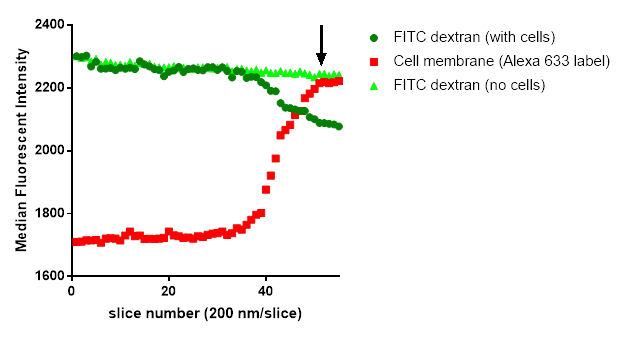


**Figure 9S.** Intensity profiles of FITC dextran along the *z*-axis measured by confocal measurements. Light green triangles correspond to intensity profiles of the bulk FITC dextran solution and dark green circles represent the intensity profile of FITC dextran within the endothelial glycocalyx (yellow dashed lines in Fig. 7S). The FITC intensity was measured in slices above the cell membrane (starting point of measurement denoted by arrow) where the glycocalyx is found.

**Table 2S:** Impact of macromolecular grafting method (non-crowded (PBS only) vs. crowded) on the protection of selected minor antigens on red blood cells^a^.

| **Antigens** | | **D** | **C** | **e** | **k** | **Fy^b^** | **JK^a^** | **JK^b^** | **Le^a^** | **M** | **N** | **S** | **S** |
| --- | --- | --- | --- | --- | --- | --- | --- | --- | --- | --- | --- | --- | --- |
| **Control** | | 4 | 4 | 4 | 3 | 3 | 4 | 3 | 2 | 4 | 3 | 4 | 4 |
| **HPG 20 kDa** | Non-crowded condition | 4 | 4 | 4 | 1 | 3 | 1 | 2 | 0 | 4 | 0 | 2 | 4 |
| **HPG 20 kDa** | crowded condition | 3 | 2 | 3 | 2 | 2 | 0 | 0 | 0 | 4 | 0 | 0 | 3 |
| **HPG 60 kDa** | Non-crowded condition | 2 | 1 | 3 | 1 | 1 | 0 | 0 | 0 | 2 | 0 | 0 | 3 |
| **HPG 60 kDa** | crowded condition | 0 | 0 | 0 | 0 | 0 | 0 | 0 | 0 | 2mf | 0 | 0 | 0 |

^a^ The phenotyping was performed at the clinical hematology laboratory in St. Paul’s Hospital in Vancouver, BC. D, C, c, E, e: Rhesus antigens; K and k: Kell antigens; Fy^a^ and Fy^b^: Duffy antigens; JK^a^ and JK^b^: Kidd antigens; Le^a^ and Le^b^: Lewis antigens; M, N, S and s MNSs antigens.

A score of 0 indicates to the absence of a particular antigen in the control RBC, whereas, a score +4 indicates a strong expression of a particular antigen on the surface of RBCs.

Results show that at identical number of HPG molecules grafted per RBC, the crowded conditions generate better protection of surface antigens against antibody recognition in comparison to the non-crowded conditions.

**References:**

1 Chapanian, R. *et al.* Enhancement of biological reactions on cell surfaces via macromolecular crowding. *Nature Communications* **5**, doi:10.1038/ncomms5683 (2014).

2 Chapanian, R., Constantinescu, I., Brooks, D. E., Scott, M. D. & Kizhakkedathu, J. N. In vivo circulation, clearance, and biodistribution of polyglycerol grafted functional red blood cells. *Biomaterials* **33**, 3047-3057, doi:S0142-9612(12)00002-6 (2012).

3 Kainthan, R. K., Muliawan, E. B., Hatzikiriakos, S. G. & Brooks, D. E. Synthesis, characterization, and viscoelastic properties of high molecular weight hyperbranched polyglycerols. *Macromolecules* **39**, 7708-7717, doi:10.1021/ma0613483 (2006).

4 Rossi, N. A. A. *et al.* Red blood cell membrane grafting of multi-functional hyperbranched polyglycerols. *Biomaterials* **31**, 4167-4178, doi:10.1016/j.biomaterials.2010.01.137. (2010).

5 Sunder, A., Hanselmann, R., Frey, H. & Mulhaupt, R. Controlled synthesis of hyperbranched polyglycerols by ring-opening multibranching polymerization. *Macromolecules* **32**, 4240-4246 (1999).

6 Leif, R. C. & Vinograd, J. Distribution of buoyant density of human erythrocytes in bovine albumin solutions. *Proceedings of the National Academy of Sciences of the United States of America* **51**, 520-&, doi:10.1073/pnas.51.3.520 (1964).

7 Levine, S., Levine, M., Sharp, K. A. & Brooks, D. E. Theory of the electrokinetic behavior of human-erythrocytes. *Biophysical Journal* **42**, 127-135 (1983).

8 Sharp, K. A. & Brooks, D. E. Calculation of the electrophoretic mobility of a particle bearing bound poly-electrolyte using the nonlinear poisson-boltzmann equation. *Biophysical Journal* **47**, 563-566 (1985).

9 Bai, K. & Wang, W. Spatio-temporal development of the endothelial glycocalyx layer and its mechanical property in vitro. *Journal of the Royal Society Interface* **9**, 2290-2298, doi:10.1098/rsif.2011.0901 (2012).

10 Bjornsson, S. Quantitation of proteoglycans as glycosaminoglycans in biological fluids using an alcian blue dot blot analysis. *Analytical Biochemistry* **256**, 229-237, doi:10.1006/abio.1997.2494 (1998).

11 Diebel, M. E., Martin, J. V., Liberati, D. M. & Diebel, L. N. The temporal response and mechanism of action of tranexamic acid in endothelial glycocalyx degradation. *Journal of Trauma and Acute Care Surgery* **84**, 75-80, doi:10.1097/ta.0000000000001726 (2018).

12 Pot, C., Chen, A. Y., Ha, J. N. & Schmid-Schonbein, G. W. Proteolytic Cleavage of the Red Blood Cell Glycocalyx in a Genetic Form of Hypertension. *Cellular and Molecular Bioengineering* **4**, 678-692, doi:10.1007/s12195-011-0180-0 (2011).

13 Ramnath, R. *et al.* Matrix metalloproteinase 9-mediated shedding of syndecan 4 in response to tumor necrosis factor alpha : a contributor to endothelial cell glycocalyx dysfunction. *Faseb Journal* **28**, 4686-4699, doi:10.1096/fj.14-252221 (2014).
